# Data on mass spectrometry-based proteomics for studying the involvement of CYLD in the ubiquitination events downstream of EGFR activation

**DOI:** 10.1016/j.dib.2018.04.049

**Published:** 2018-04-25

**Authors:** Virginia Sanchez-Quiles, Nerea Osinalde, Vyacheslav Akimov, Irina Kratchmarova, Blagoy Blagoev

**Affiliations:** Department of Biochemistry and Molecular Biology, University of Southern Denmark, 5230 Odense M, Denmark

**Keywords:** CYLD, Cylindromatosis protein, EGF, Epidermal Growth Factor, EGFR, Epidermal Growth Factor Receptor, pY, pTyr: phosphorylated tyrosine, Cbl, Casitas B-lineage Lymphoma, RTK, Receptor Tyrosine Kinase, DUB, deubiquitinase, StUbEx, Stable Tagged Ubiquitin Exchange System, SILAC, Stable isotope labeling by amino acids in cell culture, MS, mass spectrometry, LC–MS/MS, liquid chromatography coupled to tandem mass spectrometry

## Abstract

The present data article corresponds to the proteomic data of the involvement of Cylindromatosis protein (CYLD) in the ubiquitination signaling initiated by EGF stimulation. CYLD tumor suppressor protein has Lys63-chain deubiquitinase activity that has been proved essential for the negative regulation of crucial signaling mechanisms, namely the NFkB pathway. Previous results have suggested the involvement of CYLD in the EGF-dependent signal transduction as well, showing its engagement within the tyrosine-phosphorylated complexes formed following the addition of the growth factor. EGFR signaling participates in central cellular processes and its tight regulation, partly through ubiquitination cascades, is decisive for a balanced cellular homeostasis. We carried out the substitution of the endogenous pool of ubiquitin for a His-FLAG-tagged ubiquitin (Stable Ubiquitin Exchange, StUbEx), in combination with the shRNA silencing of CYLD and SILAC-labeling on HeLa cells. The subsequent tandem affinity purification of ubiquitinated proteins in control and CYLD-depleted cells was followed by mass spectrometric analysis. Therefore, we present an unbiased study investigating the impact of CYLD in the EGF-dependent ubiquitination. The data supplied herein is related to the research article entitled “Cylindromatosis tumor suppressor protein (CYLD) deubiquitinase is necessary for proper ubiquitination and degradation of the epidermal growth factor receptor” (Sanchez-Quiles et al., 2017) [1]. We provide the associated mass spectrometry raw files, excel tables and gene ontology enrichments. The data have been deposited in the ProteomeXchange with the identifier PXD003423.

**Specifications table**

**Table:** 

Subject area	Cell signaling and ubiquitination
More specific subject area	EGFR Signaling and ubiquitinome
Type of data	Mass spectrometry (MS) data
How data was acquired	MS data was acquired on a Q-Exactive mass spectrometer (Thermo Scientific).
Data format	Raw and excel files
Experimental factors	Substitution of endogenous ubiquitin for a doubly-tagged version of ubiquitin in HeLa cells, combined with silencing of CYLD using shRNA and SILAC labeling of different conditions. shControl or shCYLD cells were either left starved or stimulated with EGF for 6 min.
Experimental features	After the corresponding stimulation of shControl or shCYLD cells, protein extracts from two paralleled SILAC experiments were pooled accordingly and ubiquitinated proteins were enriched using a two steps procedure. An enrichment using Ni-NTA beads was performed, followed by immunoprecipitation of proteins using anti-FLAG specific antibodies. Enriched proteins were subjected to in-solution digestion and the resulting peptides were fractionated prior their LC–MS/MS analysis.
Data source location	University of Southern Denmark, Odense, Denmark
Data accessibility	All the data presented in this article are deposited in the ProteomeXchange consortium via de identifier PXD003423 (http://www.proteomexchange.org/). The list of the identified and quantified proteins obtained in the two replicas of the study are provided in the supplementary material of this article.

**Value of the data**

• The enrichment of CYLD within the tyrosine-phosphorylated complexes upon EGF stimulation is rapidly and strongly enhanced [2], suggesting a role for the deubiquitinase in the EGF-dependent signaling events.
• This article provides an unbiased quantitative investigation of the involvement of CYLD in the ubiquitin signaling events downstream the EGFR activation.
• The data presented here constitutes a mass spectrometry-based method for the characterization of ubiquitinated proteins in a large-scale, unbiased manner, further applicable for the elucidation of ubiquitination events beyond the EGFR pathway.
• The workflow described herein resulted in the discovery of an unexpected role of CYLD in the signaling events downstream EGF stimulation, namely that the deubiquitinase CYLD is necessary for the correct ubiquitination of the activated receptor in a counterintuitive manner.

## 1 Data

**Fig. 1:**
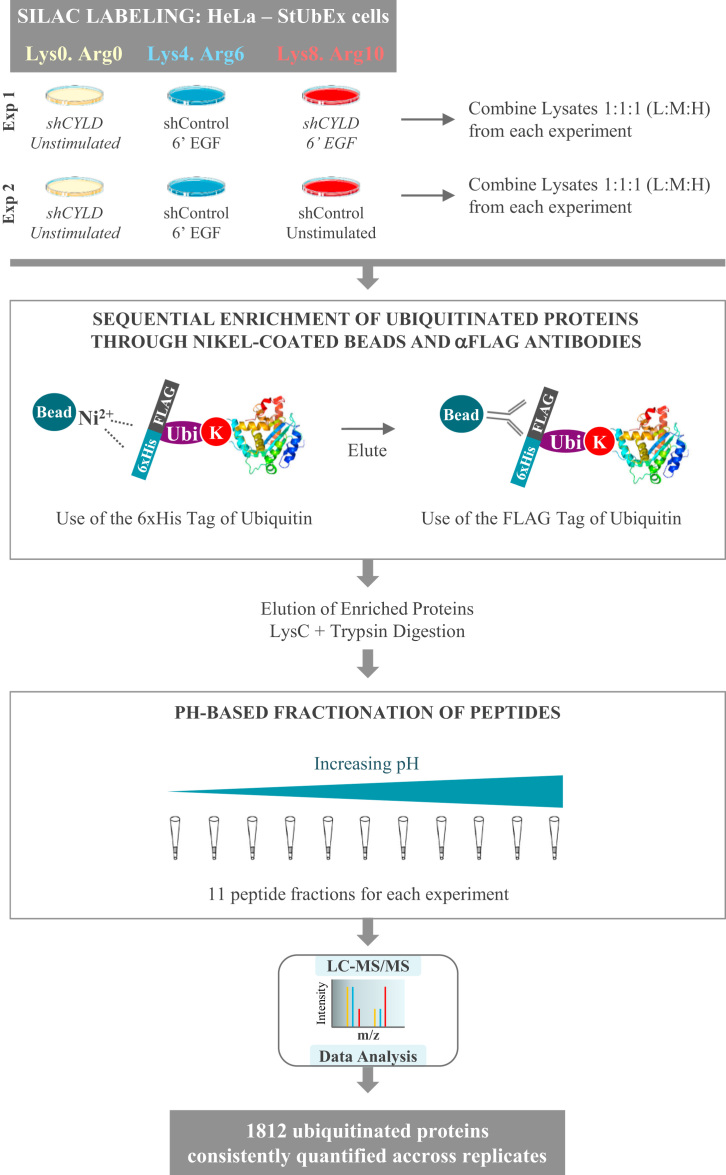
Experimental workflow for the mass spectrometry-based analysis of ubiquitinated proteins following EGF stimulation on control and CYLD-downregulated cells.

The experiments and resulting data presented here correspond to the alterations in the ubiquitinome of HeLa cells in response to EGF stimulation when the expression of the deubiquitinase CYLD is downregulated. Our interest was prompted by former findings discovering an involvement of CYLD in the EGF-dependent signaling. Although our initial reasoning was that the deubiquitinase could aid in removing ubiquitin moieties from molecular characters involved in the EGF pathway, preliminary results suggested an opposite effect, with CYLD silencing causing an impaired ubiquitination after ligand addition. We thus resolved to identify the specific proteins whose EGF-dependent ubiquitination is affected upon CYLD downregulation. Combining the StUbEx system [3] with SILAC labeling and the stable silencing of CYLD deubiquitinase, we performed a large scale, mass spectrometry-based analysis of ubiquitinated proteins upon EGF stimulation (Fig. 1) and we present herein the resulting output data.

## 2 Experimental design

In the present article we provide data resulting from the investigation of the impact of CYLD silencing in the EGF-dependent ubiquitin signaling events [1]. In order to generate the cellular system suitable for our study, several genetic engineering strategies were performed. Firstly, HeLa-StUbEx cells were transfected with a shRNA construct for the stable downregulation of CYLD expression. Next, both shControl and shCYLD cells were subjected to the doxocyclin-dependent silencing of endogenous ubiquitin moieties, that were replaced with a tagged version of ubiquitin containing tandem FLAG and 6xHistidine tags [3]. In this manner, ubiquitinated proteins could be enriched from the cellular extracts using affinity purification tools against the two tags. Finally, HeLa-StUbEx cells were metabolically labeled using SILAC for the subsequent quantitative proteomic analysis. Two biological replicas corresponding to four triple SILAC experiments were performed, so to be able to investigate all the conditions of interest (Fig. 1, representative workflow of one biological replica).

## 3 Materials and methods

### 3.1 Cell culture and assays

Human cervix epithelial adenocarcinoma HeLa cells were grown in Dulbecco's modified Eagle's medium (DMEM, Lonza) supplemented with 10% Fetal Bovine Serum, 1% Penicillin-Streptomycin and 1% l-Glutamine. For labeling experiments, cells were grown in light, medium or heavy DMEM media, containing either l-arginine (Arg0) and l-lysine (Lys0), l-arginine-^13^C_6_^14^N_4_ (Arg6) and l-lysine-^2^H_4_ (Lys4) or l-arginine-^13^C_6_^15^N_4_ (Arg10) and l-lysine-^13^C_6_^15^N_2_ (Lys8), respectively, as previously described [4], [5]. For EGF stimulation, cells were grown to a confluency of 70%, serum starved for 16 h and stimulated then with 150 ng/ml of EGF for the indicated times.

### 3.2 Plasmids and transfections

DNA constructs for RNAi silencing and generation of stable cell lines were performed as described before [3]. A lentiviral vector pSicoR (Addgene plasmid 11579) [6], which allows the selection of shRNA-expressing cells through resistance to puromicin, was used. We utilized plasmids of the 3rd generation packaging system for production of viral particles [7]: pMD2.G (Addgene plasmid 12259), pMDLg/pRRE (Addgene plasmid 12251) and pRSV-Rev (Addgene plasmid 12253), which were obtained via Addgene's Material Transfer Agreement. These DNA plasmids were kindly deposited in Addgene by Drs. Tyler Jacks and Didier Trono.

We used the following targeting sequences for RNAi: 5′-GCAATATGACGAGTTAGTA-3′ for *shControl* and 5′- GGGTAGAACCTTTGCTAAA-3′ for *shCYLD*. Verified DNA constructs were used to produce lentiviral particles as described [6] with modifications. Briefly, 10 μg of lentiviral vector and 5 μg of each packaging plasmid were co-transfected in one 15 cm dish of HEK-293T cells using the transfection reagent Metafectene (Biontex Laboratories) according to the manufacturer's instructions. Supernatants were harvested 48 and 72 h postinfection and viral particles were concentrated by ultracentrifugation. Viral stocks were diluted in cell culture media and used for infection of adherent cells to generate stable cell lines expressing the described RNAi constructs.

### 3.3 Sample preparation for mass spectrometry analysis

For the enrichment of ubiquitinated proteins, shControl and shCYLD HeLa cells containing the StUbEx construct [3] were grown in the presence of doxycycline 60 h prior the experiment. Cells were subjected to starvation in the absence of serum for the last 16 h, then left untreated or stimulated with 150 ng/ml of EGF as indicated and afterwards lysed in a 50 mM phosphate buffer pH 8.0 containing 6 M Guanidine-HCl and 500 mM NaCl. The subsequent enrichment method was as described previously [3]. Briefly, ubiquitinated proteins were enriched using Ni-NTA beads (Ni Sepharose High Performance, from GE Healthcare) and subsequently eluted with 500 mM Imidazole. A later round of enrichment was performed through immunoprecipitation using FLAG antibody –coupled beads (Sigma). Ubiquitinated proteins were eluted with TFA, reduced, alkylated and subjected to in solution digestion using LysC and Trypsin, essentially as described before [8]. The resulting peptides were fractionated through a pH gradient (pISep, Cryobiophysica), as described before [3], and purified prior to LC–MS/MS analysis.

### 3.4 NanoLC tandem mass spectrometry

Peptides were separated by reversed-phase in an EASY-nLC 1000 (Thermo Scientific) coupled to a Q Exactive mass spectrometer (Thermo Scientific) equipped with a nanoelectrospray ion source. Chromatographic separation of the peptides was performed as previously described [9] using 0.5% acetic acid as solvent A, 80% acetonitrile in 0.5% acetic acid as solvent B and an analytical in-house packed column of ReproSil Pur C_18_-AQ, 3 μm resin (Dr Maisch GmbH). The mass spectrometers were operated in positive ionization mode, in a top 12 data-dependent manner, at a resolution of 70,000 (at *m*/*z* 400) and AGC target of 1e6 for the MS survey, scanning from 300 to 1750 *m*/*z*. For the MS/MS analysis, resolution was set to 35,000 (at *m*/*z* 400), AGC target to 1e5, minimum intensity to 4e4 and isolation window to 2.0 *m*/*z*. In order to minimize the repeated fragmentation of ions, an exclusion time of 45 s was programmed. The maximum injection time values for survey and MS/MS scans were 120 ms and 124 ms, respectively.

### 3.5 Data analysis

Raw data were processed with MaxQuant [10] version 1.3.0.5, searching the peak lists against UniProt human database version 2014.01 (88479 sequence entries). Variable modifications were determined as N-terminal acetylation, methionine oxidation, deamidation of asparagine and glutamine and ubiquitination on lysine. Carbamidomethylation of cysteine was set as fixed modification. The maximum number of modifications accepted per peptide was five and the minimum peptide length for analysis was seven amino acids, with a maximum of two missed cleavages. Both peptide and protein maximum false discovery rates were set to 0.01. Supplementary table 1 contains the information related to the identified and quantified proteins obtained in the four SILAC experiments performed (two replicas). The calculation of significantly changed proteins was performed using the Perseus software [11]. The analysis of functional groups was carried out on Metacore software (Thomson Reuters). The enrichment of GO terms in the set of all quantified proteins can be found in Supplementary table 2, showing no specific bias of the StUbEx approach for any particular process, localization or molecular function.

**Fig. 2:**
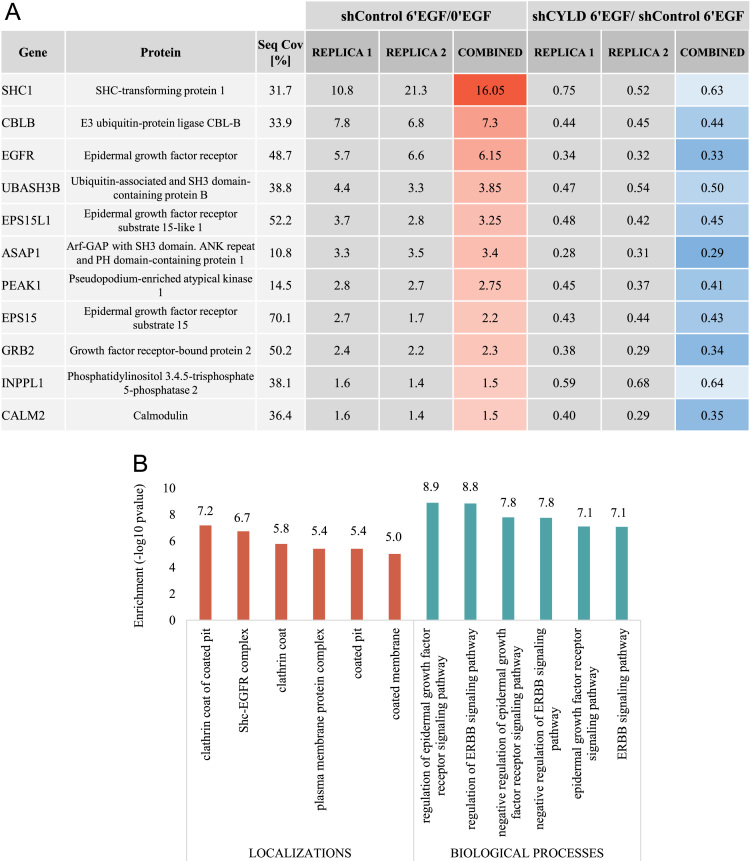
EGF-dependent ubiquitinated proteins. A: SILAC ratios of significantly enriched proteins on shControl cells upon EGF addition and their corresponding comparison with respect to shCYLD condition. B: GO terms associated to the significantly changed proteins.

**Fig. 3:**
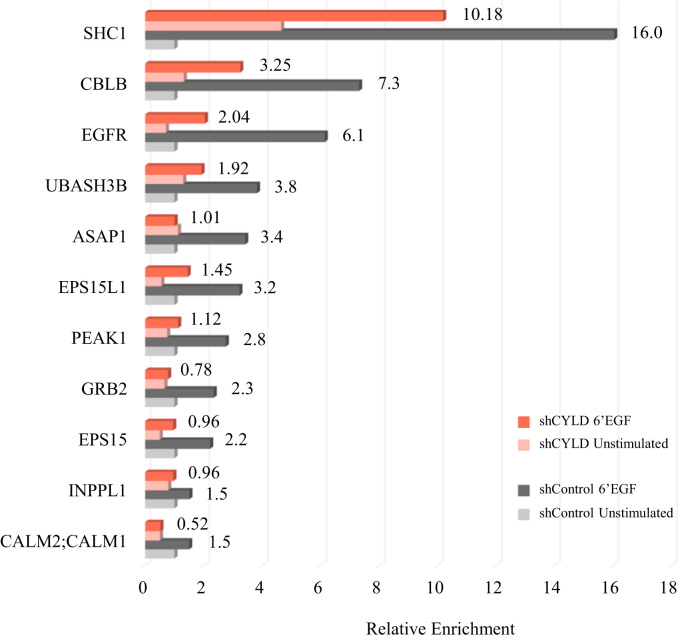
Relative enrichment of EGF-responding proteins with respect to their shControl unstimulated condition (giving the value 1).

In order to identify potential regulatory effects of CYLD in the molecular characters of the EGF pathway, we focused our analysis on the proteins which ubiquitination significantly changed in response to the growth factor in control cells and we then compared their corresponding enrichment on the silenced condition. Applying very stringent criteria (significance B, *p*<0.001 for each replica), we detected a set of 11 protein groups with a consistently increased EGF-dependent ubiquitination in control cells (see Supplementary table 3 and Fig. 2A), in agreement with previous studies defining the ubiquitinome upon EGF stimulation [3], [12]. Accordingly, their associated GO- functions and localizations are related to the EGFR/ErbB signaling pathways and the corresponding membrane structures and complexes (as shown in Supplementary table 4 and Fig. 2B). To examine the effect of CYLD down-regulation on the ubiquitination of these proteins, we compared their EGF/unstimulated ratios in control and silenced cells and found that, intriguingly, all the 11 molecular effectors display a lower EGF-initiated enrichment in the shCYLD condition. Aiming to get a clear picture of the obtained results, we translated our ratios into arbitrary units by setting a value of 1 to the shControl-unstimulated cells, thus obtaining simplified relative enrichment values (Fig. 3 and Supplementary table 3). Our data showed that most of the proteins display a similar pattern in which their ubiquitination state remains similar at the unstimulated condition, while experiencing a decrease in their EGF-induced ubiquitin attachment upon CYLD down-regulation. Due to the short time of stimulation with EGF (6 min), no changes at total protein levels could be expected. Indeed, we did not observe any detectable differences in the protein levels for EGFR and Cbl-b, which we assessed in the original research manuscript. Considering the nature and focus of our workflow, we concentrated our attention on the modified proteins. However, a discreet number of ubiquitinated sites was identified in our study and are included in Supplementary table 5.
